# A multicenter clinical nomogram for predicting post-stroke fatigue: development and validation

**DOI:** 10.3389/fneur.2026.1780370

**Published:** 2026-04-13

**Authors:** Xiaoqing Tao, Shan Wang, Li Pang, Yiling Sun, Yifei Ji, Yu Ren

**Affiliations:** 1 Department of Neurology, Beijing Anzhen Nanchong Hospital of Capital Medical University & Nanchong Central Hospital, Nanchong, Sichuan, China; 2 Department of Neurology, The Second Clinical Medical College of North Sichuan Medical College, Nanchong, Sichuan, China; 3 Department of Neurology, the First Affiliated Hospital of Chongqing Medical University, Chongqing, China

**Keywords:** LASSO, multicenter, nomogram, post-stroke fatigue, predictors

## Abstract

**Background and purpose:**

Post-stroke fatigue (PSF) is a common and disabling complication after stroke, yet its pathophysiological mechanisms remain unclear and reliable prediction tools are lacking. This study aimed to identify risk factors for PSF and develop a visualized nomogram for early prediction based on clinical and laboratory data.

**Methods:**

We conducted a retrospective cohort study of stroke patients hospitalized in the Department of Neurology at the First Affiliated Hospital of Chongqing Medical University were randomly split into training (*n* = 592) and internal validation (*n* = 254) sets. An independent cohort of 440 patients from Nanchong Central Hospital was used as the external validation cohort. Fatigue was assessed at week 4 after admission using the Fatigue Severity Scale (FSS) and Fatigue Assessment Scale (FAS). Demographic, clinical, imaging, and laboratory data were collected. LASSO regression was used for variable selection, followed by multivariate logistic regression to construct a nomogram. Model performance was assessed using the area under the curve (AUC), calibration curves, and decision curve analysis (DCA), with internal and external validation via bootstrapping.

**Results:**

A total of 846 stroke patients were enrolled and randomly split into training (*n* = 592), internal validation (*n* = 254) and external validation (*n* = 440) sets. Eight independent predictors of PSF were identified: brainstem, basal ganglia, and thalamic lesions, female sex, older age, modified Rankin Scale (mRS) score, white blood cell (WBC) count, and C-reactive protein (CRP) level (all *p* < 0.05). The nomogram showed good discrimination (AUC: 0.870, 0.862, and 0.672 for training, internal, and external validation sets, respectively), calibration, and clinical utility.

**Conclusion:**

We developed a clinically applicable nomogram based on routinely available data for early prediction of PSF. The model demonstrated good accuracy and may aid in identifying high-risk patients to guide timely intervention.

## 1 Introduction

The substantial and rising burden of stroke globally, and particularly in China, underscores the urgent need for research into stroke-related complications like post-stroke fatigue (PSF), which severely impacts the quality of life of stroke survivors (1). PSF is a common and highly disabling complication, affecting approximately 30–70% of stroke survivors (2, 3). It is characterized by persistent physical and mental exhaustion disproportionate to exertion, typically unrelieved by rest (2). PSF significantly hampers rehabilitation, reduces quality of life, and is associated with increased risks of depression, stroke recurrence, and mortality (4, 5). Despite its clinical impact, the underlying mechanisms of PSF remain poorly understood, and effective tools for early prediction and intervention are lacking. Therefore, early identification of high-risk patients is essential for optimizing management and improving long-term outcomes (6).

Previous studies have proposed several potential risk factors for PSF, including age, sex, stroke subtype, and neurological recovery (7, 8). While efforts have been made to develop predictive models, most were limited by small sample sizes, subjective variable selection, or a lack of validation, limiting their clinical utility. PSF is believed to result from a combination of mechanisms involving neuroanatomical damage, inflammatory processes, psychological factors, and functional impairment (2). However, findings have been inconsistent, and there is a lack of practical tools that integrate multidimensional data for individualized risk prediction. Furthermore, many prior studies have relied on single-center data or subjective assessment scales, which may introduce biases and limit generalizability.

The least absolute shrinkage and selection operator (LASSO) regression was employed to handle high-dimensional clinical data and mitigate multicollinearity among correlated predictors, enhancing the model’s interpretability and stability. This approach is particularly useful in medical modeling, where multiple predictors can be highly correlated (9). Nomograms were selected as the tool for risk estimation due to their practicality and ability to provide personalized, visual representations of complex models, making them highly valuable for clinical decision-making (10).

In this study, we conducted a retrospective cohort analysis using routine clinical and laboratory data from hospitalized stroke patients. LASSO regression was applied to identify significant predictors of PSF, followed by construction of a visual nomogram. Our study integrates objective, routinely available clinical parameters and employs a multicenter design, which strengthens the robustness and external validity of our findings. Our goal was to develop a robust and practical prediction model to facilitate early identification of patients at high risk for PSF and inform personalized interventions.

## 2 Materials and methods

### 2.1 Study design and participants

This retrospective cohort study aimed to develop a predictive nomogram for PSF using the LASSO regression and multivariate logistic regression models. Participants were consecutively recruited from hospitalized stroke patients admitted to the Department of Neurology at the First Affiliated Hospital of Chongqing Medical University between 01/07/2022 and 31/12/2024. Additionally, stroke patients admitted to the Department of Neurology at Nanchong Central Hospital between 08/09/2023, and 31/12/2024, were consecutively recruited as an external validation cohort. All data were accessed for research purposes on 01/05/2025.

To be included, patients were required to undergo a fatigue assessment at the fourth week after admission and provide informed consent for participation. The inclusion criteria were as follows: (1) age ≥18 years, irrespective of sex; (2) availability of all required clinical and laboratory data; (3) diagnosis of acute ischemic stroke or acute hemorrhagic stroke confirmed by either computed tomography (CT) or magnetic resonance imaging (MRI) in accordance with standardized clinical criteria; and (4) informed consent obtained from the patient or their legal guardian.

Exclusion criteria were: (1) presence of significant cognitive impairment or aphasia post-stroke that prevented effective communication; (2) a history of severe psychiatric disorders; and (3) failure to complete the required assessments within one month post-stroke.

**Figure 1:**
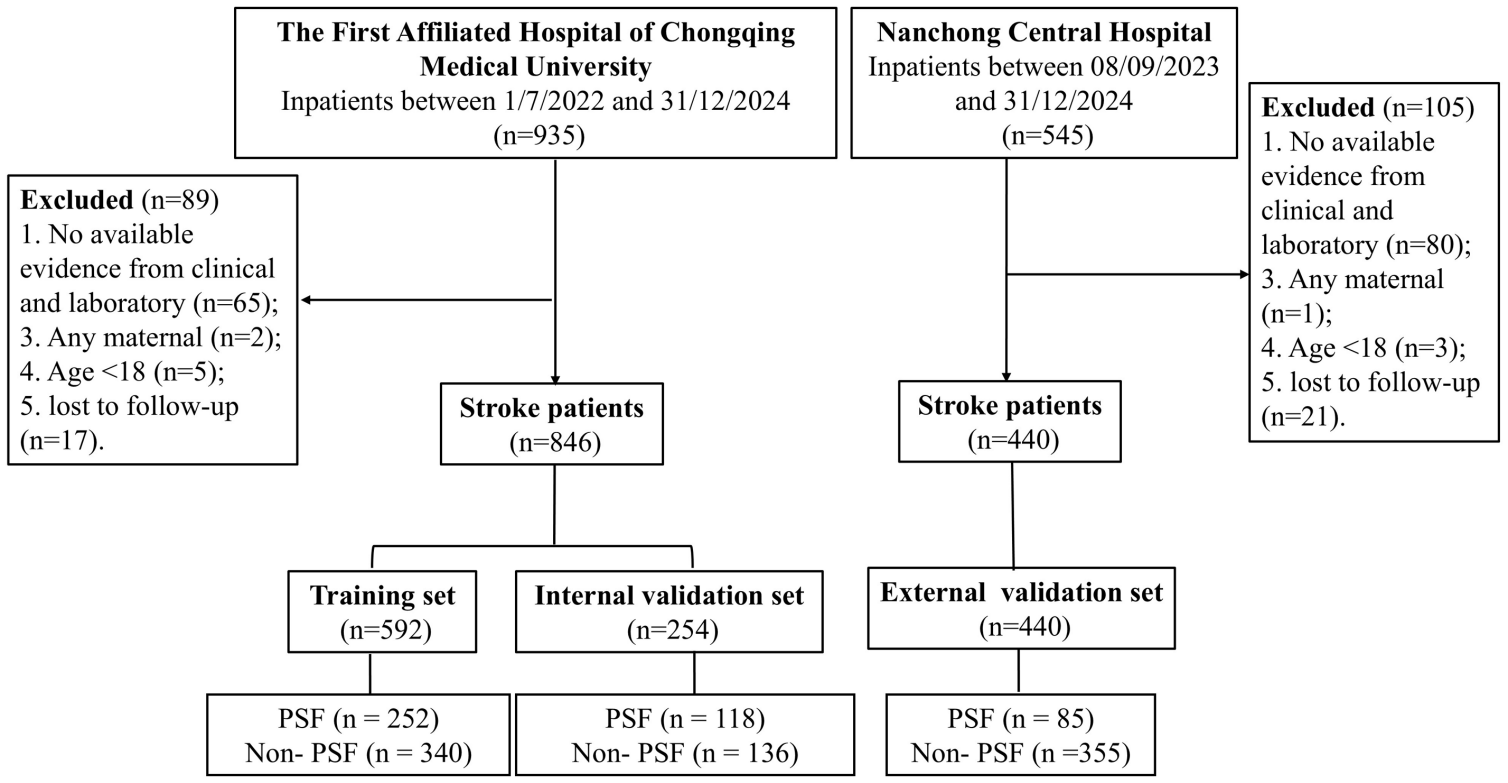
Flowchart of participant selection. PSF, post-stroke fatigue; ROC, receiver operator characteristic curve; DCA, decision curve analysis.

Initially, 935 patients were assessed for eligibility. After applying the inclusion and exclusion criteria, a total of 846 patients were included in the final analysis. These patients were randomly assigned to either a training cohort (*n* = 592) or an internal validation set (*n* = 254) using a 7:3 ratio (Figure 1). The external validation cohort consisted of 440 patients from Nanchong Central Hospital, with 85 patients in the PSF group and 355 patients in the non-PSF group. The external cohort was entirely independent of the training and internal validation cohorts.

This study was approved by the Ethics Committee of the First Affiliated Hospital of Chongqing Medical University (Ethical Approval No.: 2022-115) and conducted in accordance with the principles of the Declaration of Helsinki.

### 2.2 Data collection

Clinical data were extracted from the electronic medical records of each patient and included demographic characteristics, medical history, neurological status at admission, neuroimaging findings, laboratory test results, and clinical evaluations conducted within one month of hospitalization.

Specifically, collected variables included age, sex, clinical history (such as hypertension, diabetes mellitus, cardiovascular disease, and previous stroke), stroke-related parameters (ischemic or hemorrhagic, stroke severity, and lesion laterality), and laboratory results (complete blood count, biochemical profiles, and neuroimaging data).

Stroke severity on admission was assessed using the national institutes of health stroke scale (NIHSS), with additional NIHSS scores recorded at discharge. Functional outcome was assessed using the modified Rankin Scale (mRS) at discharge. All patients underwent CT or MRI scanning within 72 h of admission, with lesion location assessed using acute diffusion-weighted imaging (DWI) or susceptibility-weighted imaging (SWI) to enhance the accuracy and sensitivity of detecting relevant lesions.

### 2.3 Criteria for PSF

The severity of PSF was evaluated using both the Fatigue Severity Scale (FSS) and the Fatigue Assessment Scale (FAS) (11, 12). The FSS primarily measures the impact of fatigue on daily functioning, with a score of ≥4 indicating clinically significant fatigue. It provides a reliable assessment of fatigue severity and supports clinical diagnosis and management of PSF.

The FAS, consisting of 10 items, evaluates various dimensions of fatigue, with higher scores indicating greater fatigue severity. A score of ≥24 on the FAS is considered a threshold for diagnosing PSF and has been validated across 26 different medical conditions, including stroke. In our study, either a score of ≥4 on the FSS or ≥24 on the FAS was sufficient to diagnose PSF, allowing for a comprehensive assessment of fatigue severity from different perspectives. By combining both FSS and FAS, our study was able to comprehensively assess PSF severity and provide clinically meaningful data to guide therapeutic decisions.

### 2.4 Statistical analysis

Descriptive statistics were first performed for all clinical variables. Continuous variables are presented as mean ± standard deviation (SD), while categorical variables are expressed as frequencies and percentages.

LASSO regression was applied to identify key predictors of PSF by selecting the most informative variables while minimizing overfitting through cross-validation to determine the optimal penalty parameter (*λ*). Following variable selection via LASSO, a multivariate logistic regression analysis was conducted to construct the predictive model, which was subsequently visualized as a nomogram. In addition, we checked the Variance Inflation Factor (VIF) for the final set of variables to further confirm the absence of multicollinearity. No significant issues with multicollinearity were found among the selected predictors. Due to the limitations of LASSO regression in handling missing data, we excluded patients with missing values, thereby preserving model integrity and avoiding biases from imputation.

Model performance was evaluated using the concordance index (C-statistic or area under the curve [AUC]), calibration curves, and decision curve analysis (DCA) to assess clinical applicability. To validate the model and examine overfitting, bootstrap resampling was performed. Receiver operating characteristic (ROC) curve analyses were conducted using the “pROC” package in RStudio. Calibration of the model was tested via the Hosmer-Lemeshow goodness-of-fit test and calibration plots based on 1,000 bootstrap samples, with calibration quality evaluated by calibration slope (cal) and *p*-values.

DCA was conducted using the “rmda” R package to calculate the net clinical benefit of the model across a range of risk thresholds, thereby assessing the nomogram’s clinical utility. All statistical analyses were performed using SPSS version 26.0 (IBM Corp.) and R version 4.3.2.

## 3 Results

### 3.1 Study populations

Between 01/06/2022 and 31/12/2024, a total of 935 patients with stroke were screened. Of these, patients were excluded due to missing data (*n* = 65), any pregnancy-related condition (*n* = 2), age <18 years (*n* = 5), or loss to follow-up (*n* = 17). Ultimately, 846 eligible participants were included in the final analysis. These participants were randomly assigned to either the training cohort (*n* = 592) or the internal validation set (*n* = 254). In addition, 545 patients were screened at Nanchong Central Hospital between 08/09/2023 and 31/12/2024 for the external validation cohort. Of these, 105 patients were excluded due to the following reasons: missing clinical and laboratory evidence (*n* = 80), any pregnancy-related condition (*n* = 1), age <18 years (*n* = 3), and loss to follow-up (*n* = 21). As a result, 440 patients from Nanchong Central Hospital were included in the external validation cohort, with 85 patients in the PSF group and 355 patients in the non-PSF group. The external cohort was completely independent of the training and internal validation cohorts (Figure 1).

Table 1 summarizes the demographic and clinical characteristics of the entire study population. The median age of participants in the training cohort was 66.00 years (range: 37.77–88.00), while the median age in the internal validation set was 65.00 years (range: 40.00–86.67). There were no statistically significant differences (*p* > 0.05) in baseline characteristics-including demographic variables, clinical features, and available predictive factors-between the training and internal validation sets. This suggests that the random allocation of study participants into the respective cohorts was scientifically appropriate and unbiased.

**Table 1:** Baseline characteristics of subjects in the training set and validation set.

Variable	Total (*n* = 846)	Training set (*n* = 592)	Internal validation set (*n* = 254)	*p*
Demographics				
Age (years)	66.00 (38.00, 88.00)	66.00 (37.77, 88.00)	65.00 (40.00, 86.67)	0.849
Female (%)	466 (55.08)	324 (54.73)	142 (55.91)	0.947
PFS (%)	370 (43.74)	252 (42.57)	118 (46.46)	0.948
PSF using FSS cut-off (%)	330 (39.06%)	230 (38.84%)	100 (39.37%)	0.653
PSF using FAS cut-off (%)	320 (37.85%)	220 (37.17%)	100 (39.37%)	0.421
BMI (kg/m^2^)	22.84 (17.72, 29.76)	22.88 (17.74, 29.82)	22.67 (17.30, 29.35)	0.107
Smoking (%)	419 (49.53)	298 (50.34)	121 (47.64)	0.39
Alcohol drinks (%)	296 (34.99)	211 (35.64)	85 (33.46%)	0.519
Lesion sites				
Occipital lobe	50 (5.91)	37 (6.25)	13 (5.12)	0.332
Thalamus	96 (11.35)	69 (11.66)	27 (10.63)	0.537
Temporal lobe	146 (17.26)	107 (18.07)	39 (15.35)	0.754
Brainstem	83 (9.81)	58 (9.80)	25 (9.84)	1
Basal ganglia	156 (18.44)	110 (18.58)	46 (18.11)	0.631
Frontal lobe	204 (24.11)	145 (24.49)	59 (23.23)	0.811
Parietal lobe	146 (17.26)	103 (17.40)	43 (16.93)	0.566
Insular lobe	33 (3.90)	21 (3.55)	12 (4.72)	0.384
Medical History				
Hypertension diagnosis (%)	464 (54.85)	329 (55.57)	135 (53.15)	0.759
Diabetes diagnosis (%)	76 (8.98)	57 (9.63)	19 (7.48)	0.596
Previous ischemic stroke or transient ischemic attacks (%)	15 (1.77)	12 (2.03)	3 (1.18)	0.613
Chronic heart disease (%)	32 (3.78)	25 (4.22)	7 (2.76)	0.407
COPD (%)	3 (0.35)	3 (0.51)	0 (0)	0.568
Malignant neoplasms (%)	2 (0.24)	2 (0.34)	0 (0)	0.877
Admission NIHSS score	5.66 ± 5.62	4.00 (2.00, 8.00)	4.00 (2.00, 8.00)	0.873
NIHSS score at discharge	4.43 ± 6.86	2.00 (1.00, 4.00)	2.00 (0.25, 4.00)	0.848
mRS score at 4 week post-discharge	1.09 ± 1.40		1.00 (0.00, 1.00)	0.803
Hematological & biochemical findings				
WBC (10^9^/L)	7.42 (3.42, 17.61)	7.49 (3.40, 17.24)	7.31 (3.64, 17.61)	0.869
RBC (10^12^/L)	4.56 (3.28, 5.89)	4.56 (3.28, 5.88)	4.58 (3.31, 5.85)	0.705
PLT (10^9^/L)	200.00 (99.12, 399.38)	198.50 (100.55, 396.13)	203.00 (99.33, 426.15)	0.406
Hb (g/L)	138.50 (84.25, 172.88)	139.00 (90.20, 172.00)	137.00 (84.65, 173.67)	0.937
MCV (fl)	92.20 (71.61, 101.70)	92.35 (71.35, 101.70)	92.00 (74.02, 101.90)	0.500
MCHC (g/L)	331.00 (301.00, 353.00)	331.00 (300.55, 353.00)	331.00 (301.32, 349.67)	0.385
HCT (%)	41.75 (28.80, 51.79)	41.70 (28.80, 51.92)	41.80 (29.02, 51.17)	0.987
BUN (mmol/L)	5.60 (2.90, 12.97)	5.60 (2.98, 13.16)	5.50 (2.73, 12.34)	0.511
Crea (μmol/L)	71.00 (41.00, 178.88)	70.00 (40.00, 183.00)	72.50 (42.00, 166.70)	0.819
eGFR (mL/min/1.73 m^2^)	30.00 (6.00, 107.60)	30.00 (6.00, 113.00)	30.50 (6.00, 103.00)	0.908
ALT (U/L)	26.00 (8.00, 71.75)	25.50 (7.00, 64.00)	27.00 (9.32, 88.42)	0.007
AST (U/L)	25.00 (16.00, 75.00)	24.00 (16.00, 77.83)	25.00 (16.00, 71.16)	0.211
FBS (mmol/L)	6.00 (4.31, 15.78)	6.00 (4.30, 16.00)	6.00 (4.50, 14.50)	0.725
HbA1c (%)	6.00 (4.91, 12.39)	6.00 (4.90, 12.52)	6.00 (5.03, 11.93)	0.173
CRP (mg/mL)	4.63 (0.31, 26.75)	4.38 (0.31, 25.00)	5.83 (0.39, 27.00)	0.998
PCT (ng/mL)	0.03 (0.02, 0.37)	0.03 (0.02, 0.32)	0.03 (0.02, 0.37)	0.703
LDL (mmol/L)	2.56 (1.10, 4.56)	2.56 (1.14, 4.60)	2.57 (1.09, 4.45)	0.952
D2 (mg/L FEU)	0.38 (0.08, 8.08)	0.38 (0.08, 6.46)	0.38 (0.08, 11.19)	0.992
Vitamin B12 (pg/mL)	253.00 (98.12, 777.00)	257.50 (94.00, 750.25)	246.30 (107.33, 878.65)	0.462
Folate (ng/mL)	9.50 (3.50, 29.49)	9.20 (3.50, 29.32)	10.30 (3.43, 29.77)	0.144
tHcy (μmol/L)	14.20 (7.10, 39.16)	13.80 (7.10, 38.97)	15.10 (7.13, 39.21)	0.678

Data are presented as median (IQR), numbers, or percentages. BMI, body mass index; WBC, white blood cell; RBC, red blood count; PLT, platelets; Hb, hemoglobin; MCV, mean corpuscular volume; MCHC, mean corpusular hemoglobin concentration; HCT, hematocrit; tHcy, total Homocysteine; BUN, blood urea nitrogen; Crea, creatinine; ALT, alanine transaminase; AST, aspartate transaminase; D2, D-dimer; FBG, fasting blood glucose; HbA1c, glycosylated hemoglobin; CRP, C-reactive protein; PCT, procalcitonin; LDL, low-density lipoprotein; mRS, modified Rankin Scale.

### 3.2 LASSO regression for predictive variable selection

**Figure 2:**
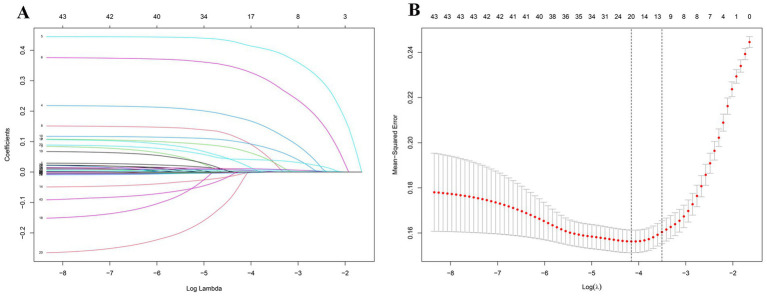
LASSO regression analysis employing tenfold cross-validation to identify predictors of PSF. **(A)** This is a coefficient profile plot created based on the log(*λ*) sequence. The *x*-axis represents the logarithm of λ, while the *y*-axis represents the regression coefficients. Each colored solid line in the graph represents a variable. As log(λ) increases, the coefficients of the variables continuously decrease, with some variable coefficients approaching zero. **(B)** Represents a 10-fold cross-validation curve for LASSO regression. The *x*-axis represents the logarithm of λ, and the *y*-axis represents the mean squared error (MSE). The dashed line on the left side of the graph indicates the λ value (0.01563987) corresponding to the minimum MSE, while the dashed line on the right side indicates the λ value (0.02999587) that is one standard deviation away from the minimum MSE. In this study, the selection of predictors is based on the λ value that is one standard deviation away from the minimum MSE (the right dashed line), where 12 non-zero coefficients were selected.

Using LASSO regression analysis, 12 variables associated with PSF were identified from the clinical and laboratory features of participants in the training cohort. These predictors included lesion location in the basal ganglia, thalamus/brainstem, cerebellum, and frontal lobe, as well as female sex, age, mRS score, white blood cell (WBC) count, blood urea nitrogen (BUN), vitamin B12, and C-reactive protein (CRP) levels (Figures 2A,B).

### 3.3 Construction of predictive model

Through both univariate and multivariate analyses, nine predictive variables were identified, as shown in Table 2. In univariate analysis, Admission NIHSS score, NIHSS score at discharge, Thalamus, Basal ganglia, Brainstem, Frontal lobe, Sex (Female), Age, mRS score at 4 weeks post-discharge, WBC count, and CRP were all significantly associated with PSF. In multivariate analysis, Thalamus (OR = 48.64, 95% CI [15.52, 220.62], *p* < 0.001), Basal ganglia (OR = 4.43, 95% CI [2.51, 7.95], *p* < 0.001), Brainstem (OR = 14.23, 95% CI [5.56, 42.34], *p* < 0.001), Frontal lobe (OR = 1.87, 95% CI [1.13, 3.12], *p* = 0.016), Sex (Female) (OR = 2.26, 95% CI [1.43, 3.61], *p* = 0.001), Age (OR = 1.06, 95% CI [1.04, 1.08], *p* < 0.001), mRS score (OR = 1.99, 95% CI [1.47, 2.73], *p* < 0.001), WBC (OR = 1.10, 95% CI [1.03, 1.18], *p* = 0.005), and CRP (OR = 1.07, 95% CI [1.03, 1.10], *p* < 0.001) remained significantly associated with PSF.

**Table 2:** Univariate and multivariate logistic regression analysis of cases and controls in the training set.

Variables	Univariate analysis		Multivariate analysis	
	OR [95% CI]	*p*-value	OR [95% CI]	*p*-value
Admission NIHSS score	1.05 [1.02, 1.08]	0.003	0.955 [0.897, 1.016]	0.148
NIHSS score at discharge	1.05 [1.02, 1.08]	<0.001	0.932 [0.868, 1.001]	0.054
Thalamus	39.86 [14.56, 164.55]	<0.001	48.64 [15.52, 220.62]	<0.001
Basal Ganglia	2.62 [1.72, 4.04]	<0.001	4.43 [2.51, 7.95]	<0.001
Brainstem	14.47 [6.60, 38.20]	<0.001	14.23 [5.56, 42.34]	<0.001
Frontal lobe	1.46 [1.00, 2.13]	0.048	1.87 [1.13, 3.12]	0.016
Chronic heart disease (%)	2.49 [1.105, 5.98]	0.032	2.74 [0.87, 8.57]	0.082
Sex (Female)	2.10 [1.50, 2.94]	<0.001	2.26 [1.43, 3.61]	0.001
Age (Years)	1.050 [1.04, 1.07]	<0.001	1.06 [1.04, 1.08]	<0.001
mRS score at 4 week post-discharge	1.50 [1.31, 1.73]	<0.001	1.99 [1.47, 2.73]	<0.001
WBC (10^9^/L)	1.14 [1.09, 1.20]	<0.001	1.10 [1.03, 1.18]	0.005
BUN (mmol/L)	1.07 [1.01, 1.14]	0.030	1.06 [0.98, 1.16]	0.166
CRP (mg/mL)	1.08 [1.05, 1.11]	<0.001	1.07 [1.03, 1.10]	<0.001

**Figure 3:**
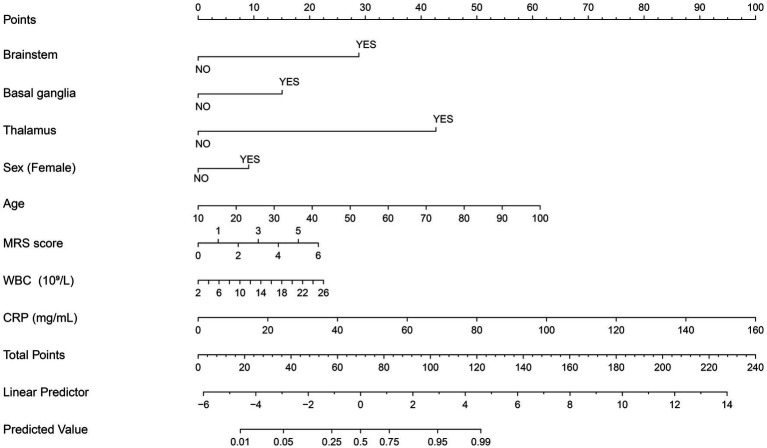
Nomogram for predicting PSF. The nomogram provides a tool for predicting the probability of post-stroke fatigue (PSF) based on several clinical variables. Each variable (e.g., brainstem involvement, sex, age, MRS score, WBC, and CRP levels) is assigned a score, and the total score is used to determine the likelihood of PSF. To use the nomogram: Locate the patient’s value for each variable on the corresponding axis (e.g., age, WBC level). Draw a line upward to determine the points associated with each value. Sum the points for each variable to obtain a total score. Use the total score to find the predicted probability of PSF on the “Predicted Value” axis. For example, a higher score on the nomogram indicates a higher probability of PSF. This nomogram can be used by clinicians to estimate a patient’s risk for PSF, aiding in early identification and potential intervention strategies. mRS, modified Rankin Scale; WBC, white blood cell; CRP, C-reactive protein.

Ultimately, eight independent risk factors for PSF were determined based on the factors present in both LASSO and multivariate regression models. These factors included brainstem, basal ganglia, thalamus, female sex, age, mRS score, WBC count, and CRP levels (*p* < 0.05). Based on these predictive factors, a nomogram was developed, as shown in Figure 3.

### 3.4 Validation of the predictive model

**Figure 4:**
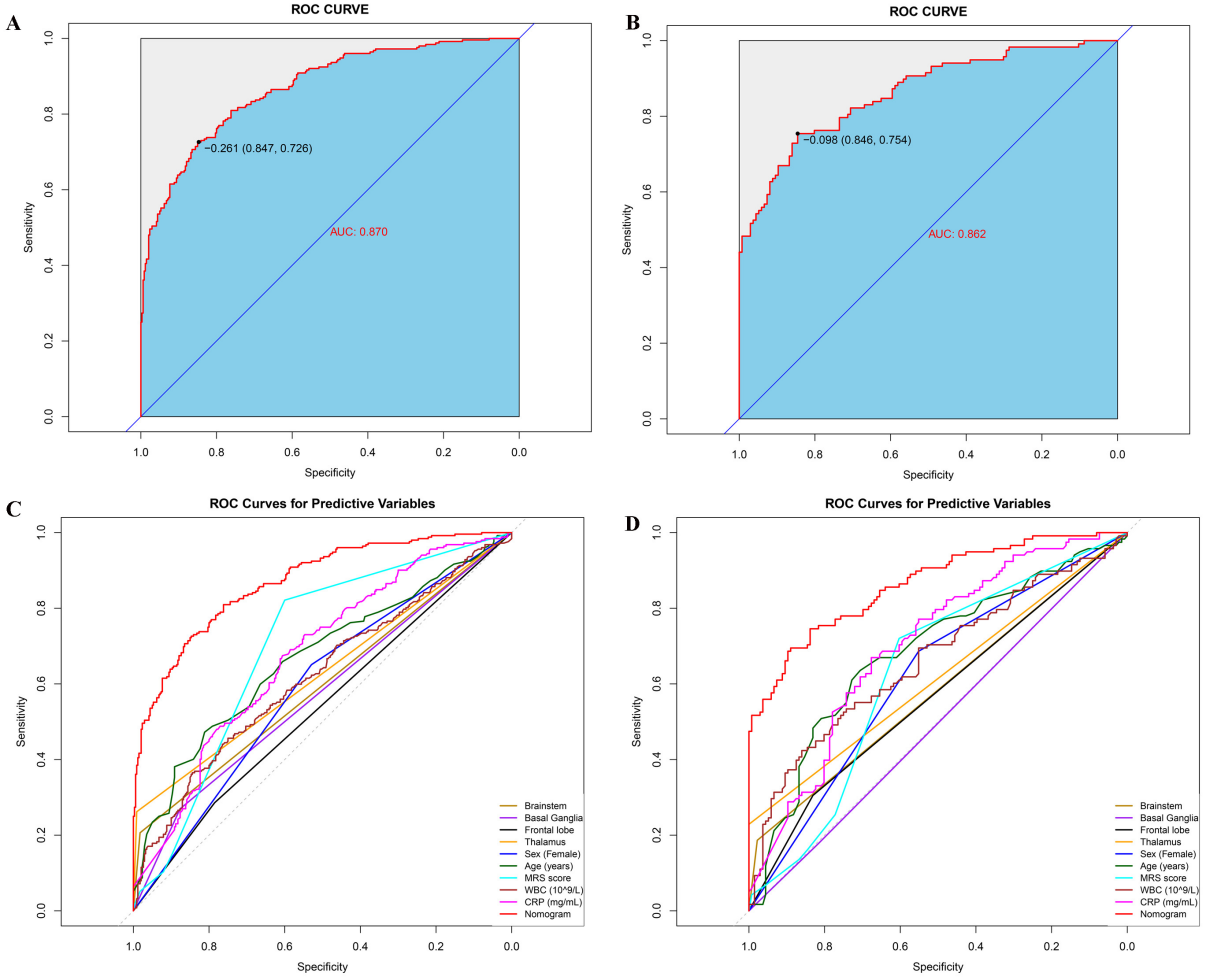
ROC of the nomogram in the training **(A,C)** and validation **(B,D)** sets. AUC, area under the curve; mRS, modified Rankin Scale; WBC, white blood cell; CRP, C-reactive protein.

Figures 4A,B show the performance evaluation of the nomogram using AUC-ROC in both the training and internal validation sets. The AUC for the training cohort was 0.870 (95% CI: 0.847–0.903), with a sensitivity of 86.5% and a specificity of 72.2%. In the internal validation set, the AUC was 0.862 (95% CI: 0.819–0.908), with a sensitivity of 89.0% and a specificity of 69.5%. Additionally, Figures 4C,D, as well as Table 3, compare the score differences between the nomogram and individual predictive variables, demonstrating that the nomogram consistently outperforms other single risk predictors in both the training and internal validation sets. These results underscore the robust predictive power of the nomogram.

**Table 3:** The value evaluation for the risk models in training and validation sets.

Variables	AUC	95%CI	Specificity	Sensitivity	Positive predictive value	Negative predictive value	*p*
Training set							
Brainstem	0.585	0.568–0.620	0.982	0.206	0.897	0.897	<0.001
Basal ganglia	0.573	0.541–0.606	0.876	0.270	0.618	0.618	<0.001
Thalamus	0.536	0.5–0.571	0.785	0.286	0.497	0.597	<0.001
Frontal lobe	0.627	0.599–0.654	0.991	0.262	0.957	0.644	<0.001
Sex (Female)	0.590	0.550–0.630	0.529	0.651	0.506	0.672	<0.001
Age (years)	0.667	0.622–0.712	0.812	0.472	0.650	0.675	<0.001
MRS score	0.700	0.659–0.740	0.6	0.821	0.603	0.819	<0.001
WBC (10^9^/L)	0.620	0.573–0.667	0.844	0.365	0.634	0.642	<0.001
CRP (mg/mL)”	0.675	0.632–0.718	0.606	0.6775	0.559	0.715	<0.001
Nomogram	0.870	0.847–0.903	0.865	0.722	0.798	0.808	Reference
Internal validation set							
Brainstem	0.582	0.545–0.620	0.978	0.186	0.88	0.581	<0.001
Basal ganglia	0.497	0.449–0.545	0.448	1.000	0.465	0.535	<0.001
Thalamus	0.568	0.516–0.620	0.831	0.305	0.610	0.579	<0.001
Frontal lobe	0.614	0.576–0.653	1	0.229	1	0.599	<0.001
Sex (Female)	0.619	0.560–0.678	0.551	0.684	0.570	0.670	<0.001
Age (years)	0.684	0.618–0.751	0.706	0.636	0.652	0.691	<0.001
MRS score	0.631	0.565–0.696	0.603	0.720	0.612	0.713	<0.001
WBC (10^9^/L)	0.663	0.595–0.731	0.860	0.424	0.725	0.632	<0.001
CRP (mg/mL)	0.704	0.640–0.767	0.676	0.669	0.642	0.702	<0.001
Nomogram	0.862	0.819–0.908	0.890	0.695	0.845	0.771	Reference
External validation set							
Brainstem	0.537	0.477–0.595	0.614	0.459	0.222	0.826	<0.001
Basal ganglia	0.504	0.446–0.563	0.608	0.400	0.197	0.809	<0.001
Thalamus	0.525	0.468–0.582	0.685	0.365	0.217	0.818	0.001
Frontal lobe	0.509	0.450–0.567	0.617	0.400	0.200	0.811	<0.001
Sex (Female)	0.500	0.441–0.558	0.675	0.457	0.193	0.807	<0.001
Age (years)	0.550	0.479–0.621	0.730	0.376	0.250	0.830	0.005
MRS score	0.613	0.556–0.670	0.803	0.424	0.340	0.853	0.004
WBC (109/L)	0.528	0.459–0.596	0.690	0.376	0.225	0.822	0.001
CRP (mg/mL)	0.533	0.484–0.623	0.839	0.282	0.296	0.830	0.006
Nomogram	0.6672	0.611–0.733	0.575	0.694	0.281	0.887	Reference

AUC, area under the curve; mRS, modified Rankin Scale; WBC, white blood cell; CRP, C-reactive protein.

**Figure 5:**
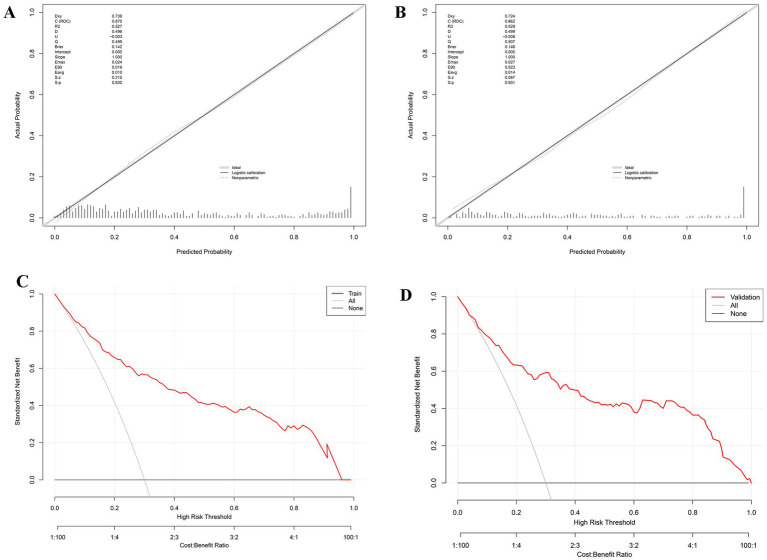
Performance of the predictive model in the external validation cohort. **(A)** ROC curve; **(B)** ROC curves for predictive variables; **(C)** calibration curve; **(D)** decision curve analysis (DCA).

The calibration curve indicated that the predicted probabilities of PSF closely matched the observed values in both the training and internal validation sets (*p* > 0.05). In Figure 5A, we show a significant consistency between the predicted probabilities of PSF and the actual observed values in the training cohort, with a mean absolute deviation (MAD) of 0.282. Similarly, in Figure 5B, the prediction in the internal validation set accurately reflected the probability of PSF (MAD = 0.307). The calibration curve evaluation demonstrated strong consistency between predicted and actual probabilities, highlighting the model’s robust predictive performance.

**Figure 6:**
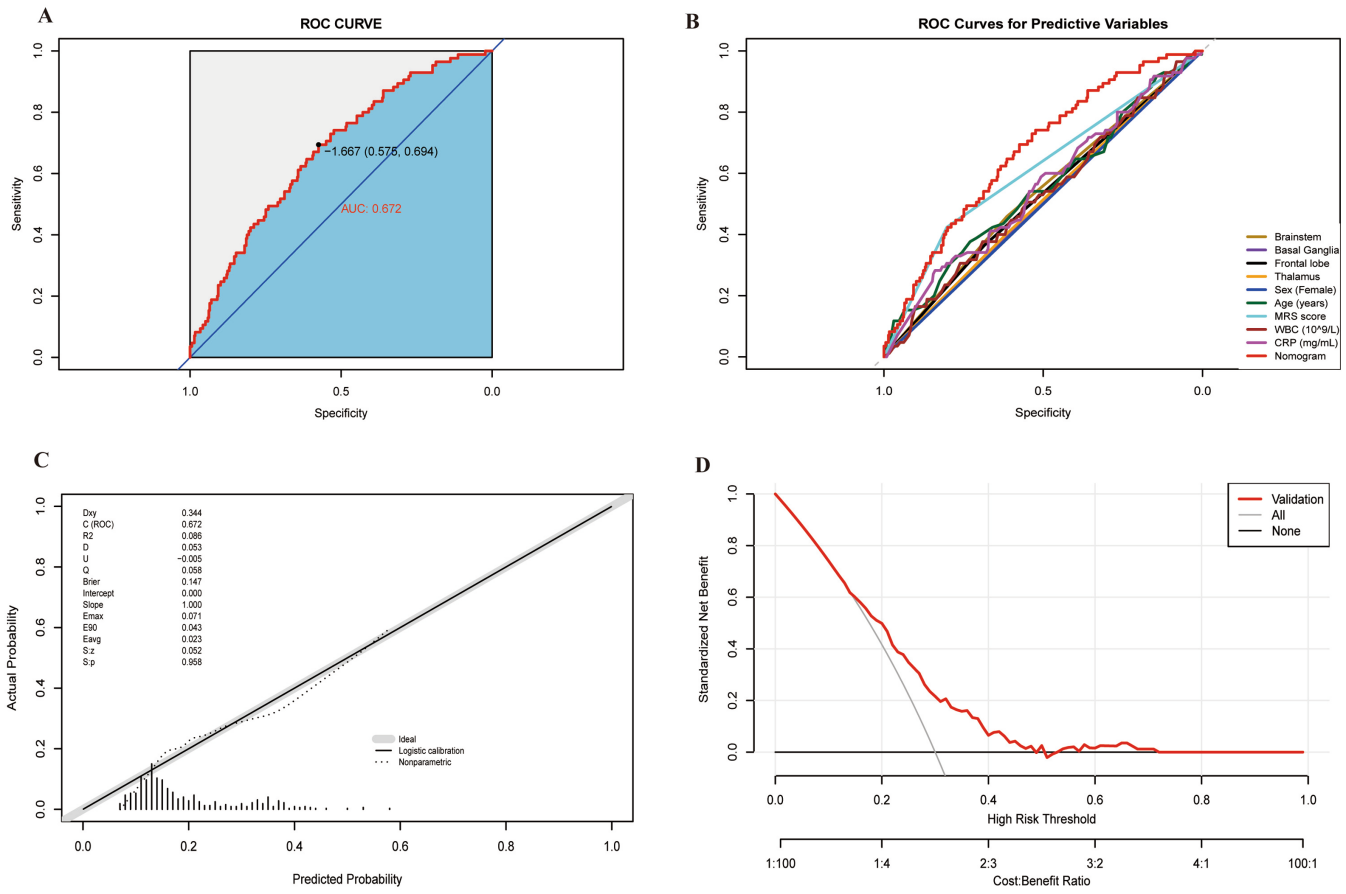
Calibration curves for the nomogram used to predict PSF risk, shown for both the training cohort **(A)** and internal validation set **(B)** (all *p* > 0.05). DCA for the nomogram used to predict PSF risk, displayed for both the training cohort **(C)** and internal validation set **(D)**. As shown in Figure, the AUC-ROC score applied to the external validation cohort confirmed that our nomogram exhibits excellent discriminatory ability in predicting the risk of PSF. The AUC-ROC value for the external validation cohort was 0.672 (95% CI: 0.611–0.733) **(A)**. The nomogram’s performance in the external validation cohort was significantly superior to that of individual predictive variables, indicating that the model outperforms other single risk prediction indicators (Table 3 and **(B)**. Furthermore, the calibration curve demonstrated precise calibration of the scoring system in the external validation cohort **(C)**, showing no significant discrepancy between the predicted probabilities and actual outcomes. Finally, the DCA curve for the external validation cohort **(D)** indicated a notable increase in net benefit within the probability threshold range of 10–50%.

However, the performance in the external validation set (Figures 6A,B) showed a significant decrease in AUC (0.672, 95% CI: 0.575–0.694), indicating moderate discriminatory ability. The calibration slope for the external cohort is 0.98, and the intercept is −0.05, showing some miscalibration due to differing post-stroke fatigue prevalence rates (Figure 6C). The DCA curves (Figures 5C,D, 6D) indicate that the model provides high net benefits in the training and internal validation sets but a more limited benefit in the external validation cohort.

## 4 Discussion

With the increasing aging population and the rising disease burden caused by strokes, PSF, a significant complication impacting patient recovery and quality of life, is receiving increasing attention. Currently, treatment and prevention strategies for PSF remain limited, and its multifactorial pathological mechanisms have not been fully elucidated. Therefore, identifying high-risk populations and developing effective risk prediction tools is crucial for personalized interventions and resource optimization.

In this retrospective two-center cohort study, we developed a nomogram to estimate PSF risk at 4 weeks after stroke using routinely collected clinical and laboratory variables. Eight predictors were independently associated with PSF: involvement of the brainstem, basal ganglia, and thalamus; female sex; older age; higher mRS score; and elevated WBC and CRP levels. These predictors collectively suggest that PSF reflects a convergence of lesion-network vulnerability, systemic inflammatory activation, and functional impairment during early recovery.

However, only a few studies have established predictive models for early identification of PSF risk. For instance, one study including patients with acute stroke developed a logistic regression model for predicting PSF risk based on clinical and psychological evaluation factors. However, its sample size was limited, and biochemical blood markers were not included, with the model’s predictive performance not systematically validated, limiting its clinical applicability and stability (13). Another study attempted to incorporate brain imaging metrics as predictors, but due to the lack of standardized tools and processes, the results showed considerable heterogeneity (14). Overall, research on PSF prediction tools is still in its early stages, and there is an urgent need to develop a personalized predictive model that combines key clinical variables, demonstrates good validation performance, and has clinical utility.

In this study, through multivariate regression analysis and LASSO regression, eight independent risk factors were identified, consistent with existing literature. Firstly, meta-analysis results indicate that lesions in specific brain regions, such as the brainstem, basal ganglia, and thalamus, are closely related to PSF occurrence (8). A study of 101 acute stroke patients, assessing PSF within 2 weeks post-stroke using the multidimensional fatigue inventory (MFI), found that lesions in the frontal thalamus/brainstem were independently associated with fatigue, with right-sided lesions significantly related to somatic fatigue (15). Research has shown an independent association between basal ganglia infarction and PSF, highlighting the potential role of this region in PSF onset. Additionally, a large voxel-based lesion-symptom mapping (VLSM) study identified that right thalamic lesions were significantly associated with fatigue symptoms 6 months post-stroke, with right thalamic lesions significantly increasing PSF risk even after controlling for confounders like age, sex, lesion volume, and depression (OR: 2.67, 95% CI: 1.46–4.88) (14). Moreover, females and older patients are more likely to experience PSF. A systematic review and meta-analysis found that female sex significantly increased PSF risk (OR 1.39, 95%CI 1.14–1.69) (8). While research results on the relationship between age and PSF vary, some studies suggest that older patients are more prone to PSF (16). However, it is important to explore potential mechanisms underlying these associations. In women, endocrine factors such as hormonal changes, including estrogen fluctuations, might increase susceptibility to PSF, and gender-related rehabilitation disparities could also contribute to these findings (17). Advanced age may be linked to a greater burden of comorbidities and decreased recovery capacity, which could amplify PSF symptoms (18).

Furthermore, indicators such as the mRS score, WBC count, and CRP levels are closely associated with PSF. In the present study, the mRS score was assessed at discharge, whereas PSF was evaluated at 4 weeks after admission. Therefore, the mRS represents an early post-stroke functional status measure that temporally precedes fatigue assessment and does not constitute data leakage. Higher discharge mRS scores may reflect greater neurological impairment and reduced functional reserve, which could predispose patients to subsequent fatigue through mechanisms such as reduced mobility, physical deconditioning, increased rehabilitation burden, and psychological stress. Consistent with our findings, previous studies have demonstrated a significant association between functional disability and PSF. A systematic review and meta-analysis found that for every one-point increase in the mRS score, the risk of PSF increases by 63% (OR = 1.63, *p* < 0.01) (8). Another prospective study found that patients with clinically significant early fatigue showed poorer improvement in functional independence during recovery. This study, using mRS to assess functional independence, showed that fatigue was associated with higher mRS scores (i.e., more severe disability) (19). These findings collectively underscore the close interplay between functional impairment and fatigue in stroke survivors. Nevertheless, given the dynamic and potentially bidirectional relationship between disability and fatigue during recovery, future longitudinal studies with repeated assessments are warranted to further clarify causal directionality. Furthermore, plasma hs-CRP levels during the acute phase of stroke were positively correlated with fatigue scores at 6 months (*r* = 0.369), with hs-CRP levels associated with increased PSF risk (adjusted OR = 3.435, 95% CI 2.22–5.31) (16). Multiple studies have found a positive correlation between hs-CRP or CRP and PSF (20, 21). Thus, hs-CRP levels appear to be a biomarker for PSF risk, though the underlying mechanisms remain unclear. Immune-inflammatory changes and secondary neurotransmitter imbalances caused by specific site damage may contribute to the pathogenesis of fatigue (22, 23). Neuroinflammation can alter neurotransmitter metabolism, disrupt synaptic homeostasis, and perpetuate sickness-behavior phenotypes, all of which may promote fatigue during recovery. Importantly, recent mechanistic frameworks emphasize the metabolic–inflammatory axis in PSF. For example, emerging work on lactylation proposes that post-ischemic metabolic dysregulation (notably lactate accumulation) can drive epigenetic and signaling changes that sustain neuroinflammatory responses and potentially contribute to persistent fatigue. Incorporating this immunometabolic perspective strengthens the biological plausibility of our biochemical findings and highlights pathways that may unify systemic inflammation with central fatigue phenotypes (24).

We developed a nomogram that integrates multiple risk factors into a quantitative tool for predicting individual PSF risk, aiding clinical decision-making. The nomogram showed strong discrimination in the derivation and internal validation cohorts (AUC ~ 0.87 and ~0.86). However, performance declined in the external validation cohort (AUC 0.672), indicating moderate discrimination and underscoring the challenges of transporting prediction models across different clinical environments. The drop in AUC is likely multifactorial. First, case-mix and spectrum effects are plausible: external cohorts often differ in stroke severity, lesion distributions, comorbidity profiles, and rehabilitation pathways, all of which can change predictor-outcome relationships. Second, there was a striking difference in apparent baseline risk between centers (fatigue prevalence approximately 44% in the primary cohort versus 19% in the external cohort). Such baseline risk shifts can produce calibration drift and degrade discrimination when the model is applied without adjustment. Third, unmeasured differences in clinical practice patterns (e.g., post-stroke rehabilitation intensity, discharge planning, follow-up adherence, and symptom reporting) may contribute to heterogeneity in PSF ascertainment and predictor effects. Collectively, these factors are consistent with the broader literature showing that predictive models often require local recalibration when deployed in settings with different baseline risks and patient compositions. Therefore, although external validation supports partial transportability, our results suggest that immediate clinical deployment in new centers should be approached cautiously.

This study has several limitations. First, although the analysis incorporated two hospitals and included an independent external validation cohort, the design remains retrospective, with inherent risks of selection bias, residual confounding, and incomplete documentation (25). Second, between-center heterogeneity-including differences in baseline PSF prevalence and case-mix-likely contributed to reduced external performance, indicating that generalizability is limited without local recalibration. Third, we did not capture key confounders strongly linked to fatigue in prior literature, particularly post-stroke depression and sleep disorders. Their omission may reduce specificity and could lead to misclassification, potentially contributing to performance degradation in external cohorts where these comorbidities may differ. Fourth, inflammatory biomarkers were measured at a single timepoint, preventing evaluation of dynamic immunometabolic trajectories that may better explain PSF persistence. Finally, by using both FSS and FAS, our study aimed to capture the multidimensional nature of PSF, increasing sensitivity but potentially leading to overestimation due to reduced specificity. Future studies should consider a composite scale or more stringent diagnostic criteria. Additionally, incorporating structured interviews, objective markers, or multidimensional scales could provide a more accurate and comprehensive assessment.

## 5 Conclusion

In summary, we developed a nomogram using routinely available variables to estimate PSF risk after stroke and performed independent external validation. While discrimination was strong in derivation/internal validation, external performance was moderate, emphasizing the real-world difficulty of transporting prediction models across heterogeneous stroke populations with different baseline risks. Transparent acknowledgment of these limitations supports the need for prospective validation and local recalibration before broad clinical adoption.

## Data Availability

The original contributions presented in the study are included in the article/supplementary material, further inquiries can be directed to the corresponding authors.
